# Clinicians’ attitudes and perceived barriers and facilitators to cancer treatment clinical practice guideline adherence: a systematic review of qualitative and quantitative literature

**DOI:** 10.1186/s13012-020-00991-3

**Published:** 2020-05-27

**Authors:** Mia Bierbaum, Frances Rapport, Gaston Arnolda, Brona Nic Giolla Easpaig, Klay Lamprell, Karen Hutchinson, Geoff P. Delaney, Winston Liauw, Richard Kefford, Ian Olver, Jeffrey Braithwaite

**Affiliations:** 1 Australian Institute of Health Innovation (AIHI), Macquarie University, Level 6, 75 Talavera Road, Sydney, NSW 2019 Australia; 2 Centre for Research Excellence in Implementation Science in Oncology, AIHI, Macquarie University, Sydney, Australia; 3 Cancer Services, South Western Sydney Local Health District Cancer Services, Sydney, Australia; 4 University of NSW, Sydney, Australia; 5 Ingham Institute of Applied Medical Research, Liverpool, Australia; 6 South Eastern Sydney Local Health District Cancer Services, Kogarah, Australia; 7 Department of Clinical Medicine, Macquarie University, Sydney, Australia; 8 University of Adelaide, Adelaide, Australia

**Keywords:** Oncology, Clinical Practice Guidelines, Guideline adherent treatment, Implementation Science, Evidence-based practice

## Abstract

**Background:**

Clinical Practice Guidelines (CPGs) synthesize the best available evidence to guide clinician and patient decision making. There are a multitude of barriers and facilitators to clinicians adhering to CPGs; however, little is known about active cancer treatment CPG adherence specifically. This systematic review sought to identify clinician attitudes, and perceived barriers and facilitators to active cancer treatment CPG adherence.

**Methods:**

A systematic search was undertaken of five databases; Ovid Medline, PsychInfo, Embase, Scopus, CINAHL, and PROQUEST. The retrieved abstracts were screened for eligibility against inclusion criteria, and a full text review was conducted of all eligible studies. Data were extracted, and a quality assessment was conducted of all included studies. The qualitative papers were thematically analyzed. Attitudes, barriers, and facilitating factors extracted from the quantitative papers were categorized within the qualitative thematic framework.

**Results:**

The search resulted in the identification of 9676 titles. After duplicates were removed, abstracts screened, and full texts reviewed, 15 studies were included. Four themes were identified which related to negative clinician attitudes and *barriers* to active cancer treatment CPG adherence: (1) concern over CPG content and currency of CPGs; (2) concern about the evidence underpinning CPGs; (3) clinician uncertainty and negative perceptions of CPGs; and (4) organizational and patient factors. The review also identified four themes related to positive attitudes and *facilitators* to active cancer treatment CPG adherence: (5) CPG accessibility and ease of use; (6) endorsement and dissemination of CPGs and adequate access to treatment facilities and resources; (7) awareness of CPGs and belief in their relevance; and (8) belief that CPGs support decision making, improve patient care, reduce clinical variation, and reduce costs.

**Conclusion:**

These results highlight that adherence to active cancer treatment CPG recommendations by oncology clinicians is influenced by multiple factors such as attitudes, practices, and access to resources. The review has also revealed many similarities and differences in the factors associated with general CPG, and active cancer treatment CPG, adherence. These findings will inform tailored implementation strategies to increase adherence to cancer treatment CPGs.

**Trial registration:**

PROSPERO (2019) CRD42019125748.

Contributions to the literature

- Implementation of Clinical Practice Guidelines (CPGs) is context specific; however, there has been no systematic assessment of the specific barriers and facilitators that apply to active cancer treatment CPG adherence.
- This manuscript systematically reviews relevant literature to identify clinician attitudes toward, and the perceived barriers and facilitators for adherence to active cancer treatment CPGs, and compares them to previously identified factors associated with general CPG adherence.
- The findings will inform targeted implementation of interventions to increase adherence to cancer treatment CPGs that overcome the context-specific barriers and utilize the identified facilitators.

## Background and objectives

CPGs synthesize the best available evidence to guide clinician and patient decision making [1]. Evidence from published clinical trials is often interwoven with clinical practice insights derived through the consensus opinion of clinical experts [2, 3]. CPGs are typically developed by government bodies or professional organizations that undertake multidisciplinary consultation, and systematic review and synthesis of the latest evidence [2]. Ideally, the evidence is explicitly linked to the CPG recommendations, and the recommendations are updated in line with the latest evidence [4].

There is a spectrum of perceptions around the utility of CPGs in medicine. CPGs are heralded as a mechanism to reduce clinical practice variation, with the aim of improving patient outcomes [2, 5]. They are viewed by some clinicians as a way of minimizing intuitive, anecdotal, and potentially biased treatment decision making [6]. Other clinicians, however, are concerned by the potential for CPGs to restrict their autonomy, and perceive CPGs as impeding their ability to tailor treatment to patients’ individual needs and preferences [7].

It has been noted that the production and dissemination of CPGs does not necessarily translate to the implementation of evidence into practice [2]. The implementation of CPGs often lags behind dissemination [8]. It has also been argued that the “uptake of research findings into routine health care is a haphazard and unpredictable process” (p. 107) [9] and barriers that impede the implementation of evidence translation arise at patient, clinician, organization, and policy levels [10].

Barriers to CPG adherence can be grouped within three domains; Clinician Awareness, Attitudes, and Work Practices regarding CPGs. A lack of clinician awareness of CPG recommendations [11] is a fundamental barrier to adherence which can be addressed by active dissemination rather than relying on simple diffusion [12].

### Negative attitudes toward CPGs also constitute important barriers

CPGs have been criticized for their focus on explicit knowledge, rather than tacit, practice-based knowledge [3]. They elicit concerns that naive prescriptive guidelines lead to “cookbook medicine” [7, 13] (p. 504) disregarding the social and organizational context of knowledge sharing, in which medicine is practiced [3]. Negative attitudes toward CPGs [14] position them as “impractical,” “rigid” tools that “reduce clinician autonomy,” that are “intended to cut healthcare costs,” while potentially increasing litigation for clinicians (p. 504) [13]. Concern that some CPGs are outdated [15] due to delays inherent in the development process, and concerns about the perceived quality of evidence underpinning CPGs [11], or use of misleading evidence, can also influence adherence [16].

Concerns have also been expressed about trial design and reporting biases [17] (with publication bias selecting for trials reporting significant results) [18], the evidence underpinning CPGs being based on clinical trials of healthier and younger patients who are unrepresentative of patients being treated in the real world (reducing CPG applicability) [19], and the influence of pharmaceutical companies on the treatment recommendations outlined in guidelines [20]. In response, there has been a call for CPG development to be more rigorous about the quality of evidence used, and to provide more refined tools to better guide implementation of recommendations [16].

### Clinician practices and care processes can influence their use of CPGs

Experienced clinicians infrequently look at CPGs, particularly for familiar procedures, and may only review CPGs before meetings to amend policies or audit practice standards [3]. To solve complex clinical problems and source up-to-date information, clinicians often use alternate trusted information sources, such as other doctors, professional networks, conferences, and medical journals and magazines, trusting the evidence, rigor, and expertise of these sources and creating internal “*mindlines*” of “*largely tacit knowledge*” (p. 1013) [3]. A lack of resources and time to implement CPGs [14], and a lack of clinician motivation or clinical inertia of practice [7], can influence adherence, as can the complexity and ease of use of CPGs [11], patient preference [11], and “*limited integration of guideline recommendations into organizational structures and processes*” (p. 213).

The successful dissemination of CPGs requires strategies that enhance CPG awareness and provide easy access to guidelines and resources [12]. Use of multifaceted implementation support strategies such as education sessions, regular prompts and reminders, engagement with local opinion leaders, and the establishment of implementation teams, which has also been successful in enhancing the implementation of CPGs [21], particularly when strategies are tailored to address identified barriers [22]. The rates of adherence to CPGs vary across cancer streams and contexts [23–25]; tailoring of CPGs, and targeting of strategies, may offer potential remedies. Factors that facilitate CPG implementation include “positive staff attitudes and beliefs, leadership support, …teamwork and collaboration, professional association support, and inter-organizational collaboration and networks.” (p. 213) [14]. A systematic review found that positive clinician attitudes to CPGs frame them as “helpful”, “educational tools”, “intended to improve the quality of care”. (p. 504) [13].

Involvement of the target group (e.g., surgeons) in CPG development has been found to enhance CPG implementation [11], and clinician age and experience also affects CPG use, with younger clinicians being more inclined to use CPGs than older or more experienced clinicians [11, 26, 27]. In addition, patient age has been found to influence the receipt of CPG adherent care for some cancer treatments, which may be related to tolerance of treatment, presence of comorbidities, or decisions regarding curative treatment [23].

In addition to the literature on CPG adherence in general, studies that have examined clinicians’ attitudes toward *cancer CPGs* have found that clinicians perceive some cancer CPGs as lacking both clarity and alternative treatment strategies that cater for a full range of patient preferences [28]. For CPGs in general, some clinicians report concerns about the quality of evidence underpinning the recommendations in general cancer CPGs [28], and the impact of these CPGs on their professional autonomy [29] and authority [30]. In addition, cancer CPGs have elicited concerns about oversimplification [30], and some clinicians simply disagree with specific cancer CPGs [28]. Other factors such as patients’ lack of health insurance [28] have been identified as a barrier to cancer CPG adherence, as has poor access to information technology (IT) or proficient IT skills [28]. It has been suggested that improved IT availability and access to CPGs via smartphone applications could facilitate use of cancer CPGs [28]. These studies also found that clinicians considered some cancer CPGs to be “convenient sources of advice,” and “good educational tools,” (p. 285) [30] that are intended to improve the quality of patient care [28, 30].

Despite this state of knowledge, there is currently a gap regarding the synthesis of clinicians’ views around adherence to active cancer treatment CPGs, and the associated barriers and facilitators to CPG adherence. There is evidence that levels of adherence across a variety of cancer treatment CPGs is relatively low [23–25, 31–38]. It is important to examine the reasons behind this, with a view to identifying potential improvements in design and content of CPGs, or their dissemination. There is evidence that CPG-adherent treatments for an array of cancers are associated with higher survival rates [33, 39–42]; however, while CPG adherence is often used as a measure of quality of care, a lack of adherence does not necessarily represent suboptimal care, if there is reasonable justification for variation [43].

Clinicians’ attitudes toward, and perceived barriers and facilitators for, adherence with *active cancer treatment specific* CPGs and CPGs *in general* are likely to overlap. However, the extent of the overlap is currently unknown. This systematic review aims to address the question: What are the attitudes of clinicians toward CPGs for active cancer treatment, and what are the perceived barriers and facilitators for adherence to these CPGs?

In this review, *barriers* refers to adherence obstacles specific to CPGs for active cancer treatment, and *facilitators* refers to enabling factors for adherence to those CPGs.

## Research design and methods

This systematic review was guided by the Preferred Reporting Items for Systematic Review and Meta-Analysis (PRISMA) statement [44], and registered on PROSPERO (2019) CRD42019125748.

### Eligibility criteria

Quantitative, qualitative, and mixed methods studies were included in the review if they reported empirical evidence, were published in English in peer-reviewed journals, and examined clinicians’ attitudes (including perceptions and views) toward, or perceived barriers and facilitators of adherence to CPGs focusing on active cancer treatment (excluding therapy with palliative intent) (Table 1). No publication date restrictions were applied. Studies were excluded if they focused on non-active cancer treatments, such as CPGs for screening, psychosocial care, symptom management, or cancer treatment with palliative intent.

**Table 1:** Eligibility criteria

Inclusion criteria:	
1. Studies must include empirical research 2. Studies must be published in English 3. Studies must be published in a peer reviewed journal 4. Studies must report treating clinician attitudes towards CPGs for active cancer treatment or perceptions of barriers or facilitators to adherence to those CPGs.	
Exclusion criteria	
Articles not including empirical research were excluded. Studies reporting on CPGs that focused on other aspects of cancer care (such as screening, psychosocial care, palliative care, or symptom management CPGs) were excluded.	

### Types of participants and outcomes

Study participants included clinicians who used cancer treatment CPGs to treat patients; this included clinicians potentially treating multiple tumor streams, such as radiation oncologists, medical oncologists, hematologists or general surgeons, and those treating single tumor streams, such as respiratory physicians. All data regarding clinicians’ attitudes or perceived barriers or facilitators to active cancer treatment CPGs were included, including qualitative, mixed methods, and quantitative data.

### Search strategy

A list of search terms (Table 2) was developed by the research team (MB, BNGE, GA) and reviewed with a medical librarian. Searches were performed in six selected databases (Ovid Medline, PsycINFO, Embase, Scopus, CINAHL, and PROQUEST) in November 2018, and repeated to update the search with current literature in November 2019. These databases were selected to enable a broad search of the literature including biomedical science, behavioral science, humanities, healthcare, and nursing literature. Four groups of search terms were combined using keywords or Mesh terms.

**Table 2:** Primary search strategy

Search terms	Limits
neoplasm* OR cancer* OR carcinoma* OR malignan* OR tumo?r* OR oncology OR metastas* AND “practice guideline” OR “clinical practice” OR “clinical protocol” OR “evidence based practice” OR “Evidence based medicine” OR guideline* OR “Practice pattern*” OR “clinical varia*” AND attitude* OR “Health personnel attitude” OR “Physician attitude” OR knowledge OR perspective* OR belief* OR barrier* OR facilitat* OR implement* OR adheren* OR concordan* OR complian* AND physician OR clinician* OR surgeon OR "medical oncologist" OR radiologist OR doctor* OR registrar* OR trainee* OR oncologist*	Title/Abstract and/or subject headings^a,b,c,d,e,f^ English^a,b,c,d,e,f^ Human^a,b,c,d,e,f^ Not (conference abstract or conference review)^b,c^ Exclude reviews ^c^ Dissertations only^a^

* Indicates truncation

^a^PROQUEST

^b^Embase

^c^Scopus

^d^Medline

^e^PsycINFO

^f^CINAHL

### Study selection

The citations and abstracts of titles identified in the searches were downloaded into Endnote and duplicates removed. The title abstracts were reviewed by four reviewers (MB, BNGE, KH, KL) to determine whether they met eligibility criteria (Table 1). Three reviewers (MB, BNGE, KH) independently conducted a blind review of a sample of 5% of title abstracts to determine inter-rater reliability. All titles were reviewed by MB, while KL, KH, and BNGE reviewed a 1%, 4%, and 5% random sample of abstracts, respectively. Any disagreements were discussed after assessment by a separate reviewer (BNGE/KH) until consensus was reached. The full texts of the included eligible abstracts were then reviewed by MB, BNGE, and KH to determine if they met the inclusion criteria. The rationale for inclusion or exclusion was recorded on a data extraction template. Reference lists of all included articles were searched for additional eligible articles.

### Data extraction

Data were extracted from all included articles using a template which included location of study, study design, sample size, data collection method (e.g., interviews, surveys), survey response rate if applicable, cancer stream and stage, discipline of participants, career experience of participants (e.g., senior clinician, registrar), description of the CPG reported, and data that related to the review question, including the key barriers to CPG adherence, key facilitators to CPG adherence, attitudes to CPGs, and other details noted as important in the study. Data were extracted from all included studies by MB, and a 5% sample of data extraction was confirmed by KH and BNGE.

### Quality assessment

Quality assessment of each included article was conducted by MB and verified by BNGE and KH using the Mixed Methods Appraisal Tool (MMAT) [45], a reliable quality assessment tool used to assess the quality of mixed studies [46], utilizing its qualitative research and quantitative descriptive research sections [45].

### Data analysis and synthesis

The included studies used a range of methodologies, including qualitative and quantitative studies, rendering results heterogenous. Due to the heterogeneity of the questions asked in the survey-based quantitative studies, statistical aggregation was not appropriate. The included articles were read multiple times until MB was familiar with the content and context of the studies, before data extraction and coding began.

#### Qualitative studies

The results section of each qualitative paper was inductively coded, line by line, by MB using NVIVO version 12 [47]. The coding involved the designation and application of summarizing labels in order to identify the meaning of text components [48]. New codes were added to the code bank as new ideas were identified [49]. After all studies were coded, an overall, refined coding framework was finalized. The initial data-driven codes were then categorized into themes that represented recurring ideas throughout the studies relating to attitudes, and perceived barriers and facilitators to CPG adherence. Themes were checked by re-reviewing the articles, to ensure they accurately represented the data [49]. Coding from a random sample of six papers was confirmed by BNGE and KH (experienced qualitative coders), to ensure that the internal validity of coding was maintained. They verified the individual codes, coding patterns, and resulting thematic framework to ensure themes were grounded in the primary data, and checked for consistency and accuracy [49, 50]. The final themes were discussed and refined by the reviewing team, resulting in a final consensus-based thematic framework.

#### Quantitative papers

Data from the results section of each quantitative study were extracted using the data extraction sheet, documenting attitudes toward CPGs or perceived barriers and facilitators to CPG adherence. The heterogeneity of these studies led to a decision to describe the data extracted, rather than attempting aggregation of results from multiple studies. The themes identified in the quantitative studies were compared with the themes identified in the qualitative papers and these were found to align. Key attitudes, barriers, and facilitators from all papers were grouped under each overarching theme.

## Results

### Search process

**Fig. 1:**
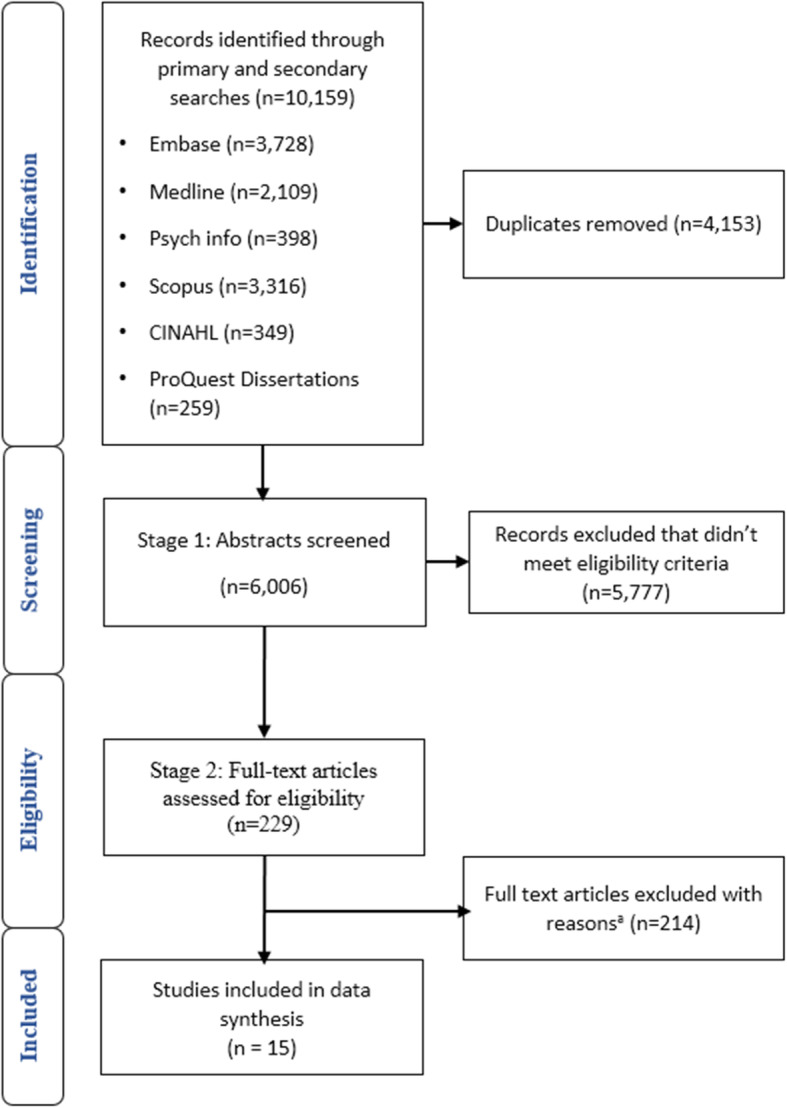
PRISMA flow diagram of search strategy [52]

The original and updated searches of the databases resulted in 10,159 title abstracts for review. Duplicate title abstracts were removed (*n* = 4153), leaving 6006 title abstracts to be screened. The level of agreement between the three reviewers (MB, BNGE, and KH) was calculated during a blinded 5% review of title abstracts: 98.4% agreement was achieved, with a Fleiss Kappa score of 0.64 [51]. KL also screened 5% of titles abstracts screened by MB, which had 99% agreement and a Fleiss Kappa score of 0.80. Of the 6006 screened title abstracts, 5777 were excluded as they did not meet the eligibility criteria, while 229 were included for full text review. Complete agreement was achieved during full text review between MB and KH, as well as between MB and BNGE, resulting in 15 studies being included in the final analysis (see Fig. 1). All included studies were assessed for quality. No papers were excluded as a result of the quality assessment, to provide a comprehensive presentation of the literature. The quality of the qualitative studies was high, while the quantitative studies were found to lack detail about nonresponse bias, and how representative the samples were of the target population (Table 3).

**Table 3:** Identified themes and quality assessment of included papers using the Mixed Methods Appraisal Tool [45]

			O'Brien (2016) [54]	Shelton (2019) [55]	Otte (2017) [53]	Brouwers (2014) [56]	Bristow (2018) [57]	Brown (2016) [58]	Carrick (1998) [59]	Fonteyne (2018) [60]	Gattellari (2001) [61]	Graham (2007) [15]	Grilli (1991) [62]	Ismaila (2018) [63]	Jagsi (2014) [64]	Ward (1997) [65]	White (2010) [66]
MMAT Quality assessment		Qualitative	✓	✓	✓	✓											
		Quantitative			✓	✓	✓	✓	✓	✓	✓	✓	✓	✓	✓	✓	✓
		Are there clear research questions?	Y	Y	Y	Y	Y	Y	Y	Y	Y	Y	Y	Y	Y	Y	Y
		Do the collected data allow to [researchers to] address the research questions?	Y	Y	Y	Y	Y	Y	Y	Y	Y	Y	Y	Y	Y	Y	Y
	MMAT–Qualitative	Is the qualitative approach appropriate to answer the research question?	Y	Y	Y	Y											
		Are the qualitative data collection methods adequate to address the research question?	Y	Y	Y	Y											
		Are the findings adequately derived from the data?	Y	Y	Y	Y											
		Is the interpretation of results sufficiently substantiated by data?	Y	Y	Y	CT											
		Is there coherence between qualitative data sources, collection, analysis and interpretation?	Y	Y	Y	Y											
	MMAT–Quantitative	Is the sampling strategy relevant to address the research question?			Y	Y	Y	Y	Y	Y	Y	Y	CT	Y	Y	Y	Y
		Is the sample representative of the target population?			Y	Y	Y	CT	Y	CT	Y	Y	N	CT	CT	CT	CT
		Are the measurements appropriate?			Y	Y	Y	Y	Y	Y	Y	Y	CT	Y	Y	Y	Y
		Is the risk of nonresponse bias low?			CT	CT	CT	CT	CT	CT	Y	CT	CT	CT	CT	CT	CT
		Is the statistical analysis appropriate to answer the research question?			Y	CT	Y	Y	Y	Y	Y	Y	Y	Y	Y	CT	Y

The randomized studies section of the MMAT the quantitative non-randomized and mixed methods sections were omitted from the table, as no studies fitted within those criteria. Y (Yes); CT (Can’t tell)

### Study characteristics

The 15 included studies comprised three interview-based qualitative studies [53–55], two studies that utilized qualitative and quantitative methods, and presented results of each method separately [53, 56], and 11 quantitative studies all using surveys [15, 57–66]. Most studies were from Australia (*n* = 5) [58, 59, 61, 65, 66] or Canada (*n* = 4) [15, 54, 56, 57]. Breast cancer CPGs were the most common focus (*n* = 6) [54, 59, 62, 64–66]. Study participants included radiation oncologists (*n* = 9), medical oncologists (*n* = 9), and surgeons (*n* = 8), with most studies including multiple professional groups (Table 4). Three studies were published in the 1990s [59, 62, 65], two in the 2000s [15, 61], and ten from 2010 to 2019 [53–58, 60, 63, 64, 66]. It is also worth noting that only one study focused on CPG implementation in low- and middle-income countries, concluding that while awareness of cancer CPGs was high among clinicians, CPG implementation was limited by inadequate facilities and CPGs that were overly complex and not applicable to the local context [63]. The remaining studies were situated in high income countries (Table 4).

**Table 4:** Characteristics of included studies

First author	Sample size	Response rate %	Qual/Quant^a^	Method	MO	RO	Surg	Urologist	Others	Cancer stream^b^	Stage	Country	CPG focus^c^
O'Brien (2016) [54]	28	68%	Qual	Interview	Y	Y	Y	-	–	Breast	Positive SLNB	Canada	cALND after positive sentinel lymph node biopsy (SLNB) regional CPG
Shelton (2019) [55]	42	–	Qual	Interview	Y	–	Y	-	Y	Colon	Stage II	USA	Adjuvant chemotherapy for colon cancer national CPGs (ASCO or the NCCN)
Otte (2017) [53]	14	–	Qual + Quant	Interview including Q card sorting	Y	–	–	-	–	Pancreatic ductal adeno-carcinoma	Locally advanced, UICC Stage III cT4N1M0	Germany	Exocrine pancreatic adenocarcinoma Treatment CPGs, including recommendations for active cancer treatment and treatment with palliative intent
Brouwers (2014) [56]	71	30%	Qual + Quant	Surveys + interviews	Y	Y	Y	-	–	NSCLC	Stage II/IIIA resected NSCLC and unresected stage IIA/B NSCLC	Canada	1. CCOPGI Chemotherapy CPG: stage II/IIIA resected NSCLC patients 2. CCOPGI Chemotherapy + radiation: unresected stage IIIA/B NSCLC patients
Bristow (2018) [57]	128	-	Quant	Survey	–	Y	–	Y	Y	Prostate	Post-Prostatectomy	Canada	ASTRO and AUA Adjuvant and salvage post prostatectomy radiotherapy CPGs
Brown (2016) [58]	157	45%	Quant	Survey	–	–	–	Y	Y	Prostate	Locally advanced	Australia	ACN Adjuvant radiotherapy treatment for prostate cancer CPG
Carrick (1998) [59]	150	64%	Quant	Survey	–	–	Y	-	–	Breast	Early stage	Australia	NHMRC Early breast cancer management CPG
Fonteyne (2018) [60]	126	18%	Quant	Survey	Y	Y	–	Y	Y	MIBC	Primary to metastatic	Belgium	Primary, Adjuvant and metastatic radiotherapy treatment for MIBC
Gattellari (2001) [61]	195	89%	Quant	Survey	–	–	Y	-	–	CRC	–	Australia	ANC/COSA CRC management CPGs
Graham (2007) [15]	520	57%	Quant	Survey	Y	Y	–	-	Y	Cancer	–	Canada	CCOPGI cancer management CPGs
Grilli (1991) [62]	770	41%	Quant	Survey	Y	Y	–	-	Y	Breast, CRC, Ovarian	–	Italy	Italian National CPGs for Breast, CRC, and ovarian cancer treatment
Ismaila (2018) [63]	101	53%	Quant	Survey	–	Y	–	-	–	Cancer	–	Nigeria	International, national, local oncology CPGs
Jagsi (2014) [64]	896	60%	Quant	Survey	Y	–	Y	-	–	Breast	–	USA	NCCN CPG for Breast cancer
Ward (1997) [65]	69	77%	Quant	Survey	Y	Y	Y	-	Y	Breast	Early stage	Australia	NHMRC early breast cancer CPG
White (2010) [66]	63 (survey)	58%	Quant	Survey + Patterns of care study	–	–	Y	-	–	Breast (DCIS)	DCIS	Australia	Australian treatment recommendations for DCIS

^***a***^*Qual* qualitative research, *Quant* quantitative research, *MO* medical oncologist, *RO* radiation oncologist, *Surg* surgeon

^**b**^*cALND* completion axillary lymph node dissection, *CRC* colorectal cancer, *DCIS* ductal carcinoma in situ, *MIBC* muscle invasive bladder cancer, *NSCLC* non-small cell lung cancer

^**c**^*ACN* Australian Cancer Network; *AUA* American Urological Association; *ASCO* American Society of Clinical Oncology; *ASTRO* The American Society for Radiation Oncology, *CCOPGI* Cancer Care Ontario, Practice Guideline Initiative; *COSA* Clinical Oncology Society of Australia; *NCCN* National Comprehensive Cancer Network; *NHMRC* National Health and Medical Research Council; *SLNB* sentinel lymph node biopsy

### Themes

Four themes regarding negative *attitudes and barriers* to active cancer treatment CPG adherence were identified (themes 1–4), and four separate themes classified positive attitudes and *facilitating factors* to active cancer treatment CPG adherence (themes 5–8). Table 5 presents the themes, the proportion of clinicians reporting each factor, and the tumor stream focus.

**Table 5:** Identified Themes and Subthemes and the proportion of clinicians reporting each subtheme

Included studies	O'Brien (2016) [54]	Shelton (2019) [55]	Otte (2017) [53]	Brouwers (2014) [56]	Bristow (2018) [57]	Brown (2016) [58]	Carrick (1998) [59]	Fonteyne (2018) [60]	Gattellari (2001) [61]	Graham (2007) [15]	Grilli (1991) [62]	Ismaila (2018) [63]	Jagsi (2014) [64]	Ward (1997) [65]	White (2010) [66]
Qualitative methods	✓	✓	✓	✓											
Quantitative methods			✓	✓	✓	✓	✓	✓	✓	✓	✓	✓	✓	✓	✓
Cancer Stream	Breast	Colon	Pancreatic	NSCLC	Prostate	Prostate	Breast	MIBC	CRC	Cancer	Breast, CRC, ovarian	Cancer	Breast	Breast	Breast
**Theme 1 Barriers: concern over CPG content and currency of CPGs**		✓	✓	✓		✓	✓		✓	✓		✓	✓	✓	
Some CPG recommendations are biased				✓											
CPG are not always applicable to specific settings or feasible							25%					✓			
CPGs are not always clear			✓											25%	
CPGs can be hard to read														17%	
Outdated CPGs, or slow to be updated		✓								31%			6%		
Some CPGs are perceived to be cookbook medicine that oversimplifies difficult or controversial treatment decisions			✓			28%	46%		45%	13%			24%	26%	
Some CPGs are too complicated or complex to follow												✓	23%		
Some CPGs are too rigid to apply to practice				✓		31%				7%			20%		
CPGs do not always take into account patient preferences or circumstances						18%			37%						
Concerned that some CPGs were developed by people who were not engaged with clinical practice														6%	
Concerned that CPGs are intended to cut costs						12%			17%						
**Theme 2 Barriers: concern about the evidence underpinning CPGs**	✓	✓	✓	✓	✓	✓			✓	✓			✓	✓	
CPGs underpinned by controversial evidence or a lack of evidence	✓				✓	30%									
Clinical trial patient populations that does not contain patients that clinicians routinely see			✓	60%											
The existence of contradicting CPGs or CPGs that provide contradicting or controversial recommendations or advice		✓				16%				10%					
Some clinicians prefer their own interpretation of the evidence over the synthesis of evidence in particular CPGs													30%		
CPGs do not always take into account clinical experience									36%					10%	
**Theme 3 Barriers: clinician uncertainty and negative perceptions towards CPGs**	✓		✓	✓		✓	✓	✓	✓	✓		✓	✓	✓	
Clinical equipoise and practice habits that differ to the CPG recommendations				✓		✓				3%					
Concerns about side effects associated with CPG recommendations or past experience of patient adverse effects from CPG recommended treatments	✓			3%		25%		10%							
Limited medical expertise to implement the CPG recommendation				10%											
Clinician subjectivity’ regarding specific treatments and a perception that the CPG recommended treatments are not necessarily appropriate for specific patients	✓		✓												
A lack of awareness of CPGs								12%				✓			
CPGs challenged clinician authority or autonomy						15%				8.5%			20%		
Some CPGs limit the application of clinical judgement						18%									
Clinicians disagreeing with specific CPG recommendations							✓	2%							
Limited experience with CPG recommended treatments								14%							
A lack of outcome expectation of the CPG recommendations				67%		70%		10%							
Concerned that CPGs will expose them to litigation issues							37%		33%					45%	
**Theme 4 Barriers: organizational and patient factors**	✓			✓		✓		✓				✓			
Limited access to treatment services				✓								✓			
Treatment referral processes that are slow				31%											
Referral processes that are unreliable				10%											
Referral processes that are complex				12%											
Surgeons’ hesitancy to refer patients to other clinicians				13%											
A lack of support from organizational and clinical leadership				32%											
CPG recommendations are not always cost effective				8%											
Patient preferences regarding treatment choice	✓			✓				< 1%							
Patient comorbidities and tumor specific characteristics	✓														
The level of family support available to patients, and access to transport influences the treatment provided				✓											
family perceptions of or experiences of treatments were found to influence patient attitudes				✓											
The age of the patient				✓											
Concerns about costs of treatments or concern that adhering to CPG will increase healthcare costs, and other external barriers						61%		18%							
Poor accessibility to CPGs						22%						✓			
**Theme 5 Facilitators: CPG accessibility and ease of use**	✓			✓			✓		✓	✓		✓		✓	
Having highly skilled clinicians with adequate expertise to implement the CPG is important				✓											
CPGs should be treated as guides, not rules, to cater to individual patient needs	✓														
Some CPGs are considered good summaries of up-to-date evidence				✓			97%								
Clinicians felt it was important that CPGs were updated regularly	✓														
Some CPGs are considered easy to understand							96%								
Some CPGs are considered flexible										67%					
Some CPGs are considered implementable										87%					
User-friendly formats were considered a strength of CPGs														83%	
Some CPGs are developed in a timely manner										46%					
Adapting and revising CPGs to cater for local needs, and holding meetings about the revised CPG was an important factor									63%			16%		76%	
Access to and availability of IT technology that integrates CPGs into the software used to record and order treatments, and provides feedback to clinicians is important														50%	
**Theme 6 Facilitators: endorsement and dissemination of CPGs along with adequate resources**				✓				✓	✓			✓		✓	
Clinician and clinical organizational support is important				✓											
Collaboration between clinical disciplines in Multi-Disciplinary Teams (MDTs) is important				✓											
Easy access to treatment services for patients is important				✓											
CPG dissemination via medical college programs is important														84%	
CPG endorsement by government research organizations is important														83%	
CPG endorsement by medical colleges is important									74%					86%	
Recommendations by respected peers, or discussions with respected peers is important									51%					71%	
Symposia about CPGs are important								47%	74%						
Provision of emails or websites that summarized updated CPGs, or current clinical trials underpinning CPGs are important								54%							
Access to treatment facilities and adequate resources to implement CPGs is important									46%			22%			
Audits and feedback are important									54%						
Multidisciplinary clinical care pathways or MDT discussions increase awareness of CPGs								52%	47%						
**Theme 7 Facilitators: awareness of CPGs and belief in their relevance**	✓	✓	✓	✓	✓	✓		✓	✓	✓	✓	✓	✓	✓	✓
High clinician awareness of CPGs	✓	✓	✓		49-82%	54%		✓	86%	83%	44-60%	✓	74%	80%	76%
Agreement with and support for CPG recommendations	✓			✓							40-93%		71%		49%
Confidence in CPGs was high when the guidelines were considered high quality										85%					
Use of or compliance with CPGs was generally reported to be high	✓				✓	78%		5-68%	55%	44%		93%	24-48%	39%	
CPGs should be “developed by credible individuals” and include lists of CPG committee members should be published										93%				75%	
Financial disincentives for surgeons who do not follow the guidelines									38%						
**Theme 8 Facilitators: CPGs support decision making, improve patient care, reduce clinical variation and reduce costs**	✓	✓	✓	✓		✓	✓		✓	✓			✓	✓	
CPS are good, convenient sources of advice or information with unambiguous recommendations				✓		89%			79%	94%			98%	88%	
CPS are considered to be good, useful and educational tools for making treatment decisions that help clinicians orientate treatment decisions		✓	✓	✓		89%			84%	98%			99%	90%	
CPGs help decision making during treatment complications, to double check treatment decisions, especially when clinicians don’t do not have access to MDTs and are clinically useful	✓								59%	89%					
CPGs reduced practice variation and increased the uniformity of care across disciplines	✓														
CPGs help clinicians and patients to reach agreement							86%								
CPGs increased the confidence of clinicians									64%						
Support clinicians’ legal defense when they are adhered to							41%		54%					42%	
CPG recommendations are balanced in terms of harms and benefits										59%					
A “multidisciplinary focus” is important in CPGs														94%	
Not being prescriptive is considered a strength of CPGs														59%	
CPGs are part of routine practice							97%								
CPGs improve patient wellbeing							80%								
CPGs improve patient survival, outcomes and quality of care						52%	47%							46%	
CPGs are intended to enhance the quality of patient care						89%				95%			98%		
CPGs are intended to minimize healthcare costs													51%		

## Negative attitudes and barriers

### Theme 1: concern over CPG content and currency of cancer treatment CPGs

Clinicians reported that some CPGs are not always applicable to specific settings [63], are not clear [65], are hard to apply [53], and hard to read [65]; all potential barriers to CPG adherence. It should be noted that one study [53] referred to CPGs for the treatment of locally advanced, UICC stage III cT4N1M0 pancreatic ductal adenocarcinoma, with recommendations for palliation as well as active cancer treatment. Clinicians thought some CPGs were slow to be updated [15], or were outdated [55, 64]. Across five studies, a range of clinicians perceived that some CPGs can promote “cookbook medicine” [15, 58, 59, 61] (p. 150) [64], that is generic [53], and can oversimplify difficult or controversial treatment decisions [65].

Guidelines are very generic, which means they address certain age groups or patients that have benefited from a certain type of chemotherapy in a certain way. And this does not cover all the different factors …, like patient preferences or social environment, sometimes the guidelines cover the age, but overall it is all very simplified. Otte (2017) (p. 784) [53]

Other barriers to adherence included CPGs being too complicated or complex to follow [63, 64], or that the recommendations were not feasible [59]. In three studies, clinicians felt that some CPGs were too rigid to apply to practice [56, 58, 64]; however, the majority of clinicians in another study disagreed with that sentiment [15]. A small proportion of clinicians surveyed in one study felt that recommendations in a cancer CPG may be biased, which could also limit adherence [56].

Few clinicians agreed that CPGs take into account patient preferences or needs [58] or individual circumstances of patients [61]. A small number of clinicians were concerned that some CPGs were developed by people disengaged with clinical practice [65], and clinicians in two studies felt that CPGs were intended to cut costs [58, 61].

### Theme 2: concern about the evidence underpinning cancer treatment CPGs

Clinicians raised concerns about the uncertainty generated by CPGs that contradict each other [55, 58] and felt that this contributed to the complexity of inter-disciplinary decision making about treatment [54]. Clinicians also believed that some CPGs were underpinned by controversial [15, 54] or conflicting evidence [57], or a lack of evidence [58], which could also act as a barrier to adherence. Some clinicians in another study preferred their own interpretation of the evidence over the synthesis of evidence in particular CPGs [64]. Concerns were also raised that clinical trial patient populations from the studies underpinning some CPGs were not representative of the patients that clinicians routinely see [53, 56]. Some clinicians felt that CPGs did not take into account clinical experience [61] and “emphasized published evidence to [the] detriment of clinical judgment” (p. 363) [65].

The patients who present in real life are much more variable with respect to functional status and comorbidities than the stage IIIA/ IIIB patients reflected in the evidence and PG recommendations. This lack of connection between the real life patient and the study patient can undermine the value, relevance and utility of the [Practice Guideline]. Brouwers (2014) (p. 43) [56]

### Theme 3: clinician uncertainty and negative perceptions of cancer treatment CPGs

A few clinicians felt that CPGs challenged their authority [15] and autonomy [58, 64] by limiting their application of clinical judgment [58]. Clinical equipoise and habits that differed from the CPG recommendations were suggested barriers to CPG adherence [56, 58] and a small number of clinicians in one study felt that implementing a specific CPG would require too many changes to their practice [15]. Some clinicians reported disagreeing with specific CPG recommendations [59], and a minority felt that disagreeing with a CPG could be a barrier to adherence [60], noting that this study [60] did not differentiate between attitudes toward CPGs for radiotherapy in the primary, adjuvant, or metastatic (and potentially palliative) settings. A lack of awareness of CPGs was reported as a barrier by a small number of clinicians in two studies [60, 63].

A small number of clinicians raised clinician subjectivity regarding specific treatments for particular patients as a potential barrier to CPG adherence; some CPG-recommended treatments were perceived to be inappropriate for specific patients [53, 54].

The concrete treatment recommendation physicians make to an oncologic patient depends highly on their subjective estimation of the patient’s biological age and prognosis. Clinical guidelines are seen as an important point of reference, but cease being helpful in highly individual cases. Otte (2017) (p. 784) [53]

Some clinicians in three studies felt that the risk of side effects as a result of adhering to the CPG-recommended treatment was a barrier [56, 58, 60], as well as limited medical expertise or clinician skill [56], or limited experience with the recommended treatment [60]. A lack of expectation of improved patient outcomes as a result of adhering to CPGs was another potential barrier reported in three studies [56, 58, 60]. A significant proportion of clinicians in three studies were concerned that CPGs could expose them to litigation [59, 61, 65], although some clinicians felt that CPGs would also protect them [59].

### Theme 4: organizational and patient factors

A multitude of organizational barriers to CPG adherence were identified: limited access to treatment facilities and services [56, 63]; treatment referral processes that are slow, unreliable, and complex [56]; and a lack of support from organizational and clinical leadership [56]. A small proportion of clinicians also felt that surgeons’ hesitancy to refer patients to other clinicians (like medical or radiation oncologists) was a barrier [56]. The costs of treatments was raised as a barrier in one study [60], while clinicians in other studies expressed concern that adhering to CPGs would increase healthcare costs [58] or that CPG recommendations were not always cost effective [56]. Poor access to CPGs in general was also identified as a factor that could limit CPG adherence [58, 63].

Patient preferences regarding treatment choice were perceived to limit adherence to CPG recommendations where these differ from CPG recommendations [54, 56, 60]. Patient comorbidities and tumor-specific characteristics were also found to limit clinicians’ adherence to CPG recommendations if they perceived the treatments to be inappropriate [54]. The level of family support available to patients and patient access to transport were found to influence the treatments that clinicians offer [56], and family perceptions and experiences of treatments influenced patient attitudes [56]. The age of the patient was also mentioned as an influence on clinicians’ choice of treatment in one study [56].

## Positive attitudes and facilitators

### Theme 5: cancer treatment CPG accessibility and ease of use

Theme 5 included factors that were seen to facilitate adherence to CPG recommendations. Clinicians were generally positive about cancer treatment CPGs, finding them easy to understand [59], flexible, and implementable [15]. CPG user-friendly formats were considered a strength of CPGs [65]. Having highly skilled clinicians with adequate expertise to implement a CPG was seen as important [56]. Clinicians felt that CPGs should be considered as guides, not rules, to allow flexibility to cater to individual patient needs [54], and they should contain up-to-date evidence [56] and be updated regularly [54]. Specific CPGs were considered applicable by a large proportion of clinicians in one study [56].

Guidelines, by definition, are simply guides, they are not protocols.’ (S2) ‘The guideline is not a cookie-cutter for every patient.’ (S11). O'Brien (2016) (p. 129) [54]

Many clinicians thought that specific CPGs were a good summary of the latest evidence [15, 59, 65] and had been developed in a timely manner [15] while other CPGs were seen as providing an “unbiased synthesis” of the underpinning evidence [58, 61] (p. 151). It was considered important that CPGs cited the strength of evidence underpinning the recommendation [61, 65]. Clinicians in one study were positive about the evidence underlying a specific CPG, finding the evidence base “complete,” “convincing,” “informative,” “relevant,” “strong,” and “current.” (p. 40) [56]. The majority of clinicians in another study valued CPGs that were based on randomized control trials and that provided detailed recommendations, preferring 9–10 years of follow-up evidence to convince them of the benefit of specific treatment options [58].

Adapting and revising CPGs to cater for local needs was an important factor that was seen to influence implementation and adherence [61, 63, 65] and holding meetings to locally adapt a CPG was considered an effective implementation strategy [61]. Access to, and availability of, IT technology that integrated CPGs into the software used to record and order treatments and provide feedback to clinicians was also reported to be an important implementation strategy [65].

### Theme 6: endorsement and dissemination of cancer treatment CPGs along with adequate access to treatment facilities and resources

Most clinicians in one study reported that CPG dissemination via medical college programs, or other education related programs [65], as well as endorsement by government research organizations [65] or medical colleges [61, 65] were important strategies facilitating CPG adherence. Recommendations by respected peers [65], discussions about CPGs [61], and CPG symposia [60, 61] were also considered important facilitators.

Many clinicians suggested that the provision of emails or websites that summarized updated CPGs or current clinical trials underpinning CPGs were potential facilitators to enhance awareness of CPGs [60]. Access to treatment facilities with adequate resources to implement a CPG was identified as an effective facilitator to CPG use [56, 61, 63], as was audit and feedback [61]. The presence of clinician and clinical organizational support were identified as facilitating factors of CPG adherence [56]. Multidisciplinary clinical care pathways [61], multi-disciplinary team meeting (MDTM) discussions [60], and collaboration between clinical disciplines in multi-disciplinary teams (MDTs) were suggested as ways to increase awareness of CPGs and support the decision making process [56].

### Theme 7: awareness of cancer treatment CPGs and belief in their relevance

The vast majority of clinicians reported being aware of the CPGs each study focused on [54–56] with some variation in awareness [15, 57, 58, 60–66]. In one study, awareness of CPGs was found to vary across disciplines, with radiation oncologists more aware of specific radiation therapy guidelines than urologists [57], and increasing clinician awareness of CPGs was identified as a facilitator to increase CPG usage (in low income countries) [63]. Agreement with CPG recommendations varied but was generally high [62, 64, 66] and support for CPG recommendations was considered an important factor for adherence [56]. Confidence in CPGs was high when the guidelines were considered high quality [15]. Use of or compliance with CPGs was generally reported to be high [15, 57, 58, 60, 63–65].

In one study, clinicians reported a variety of attributes of CPGs to be important, including the quality and level of evidence underpinning the CPGs, the “specification of the patient population to which a guideline is most applicable,” the “strength of the recommendation,” and the provision of cost effectiveness data (p. 151) [61]. Clinicians felt that CPGs should be “developed by credible individuals,” (p. 611) [15] and that lists of CPG committee members should be published [65]. Some clinicians in one study felt that financial disincentives for surgeons who do not follow the guidelines would be effective strategies to facilitate adherence [61].

### Theme 8: cancer treatment CPGs support decision making, improve patient care, reduce clinical variation, and reduce costs

CPGs were considered to be good, useful, and educational tools for making treatment decisions by most clinicians [15, 55, 56, 58, 61, 64, 65].

Despite differences in reported use, most providers agreed that due to uncertainty regarding the benefits of [Adjuvant Chemotherapy] for this patient population, guidelines are important to help patients understand treatment options and to help providers make the most appropriate recommendation. Shelton (2019) (p. 287) [55]

CPGs were also considered to be “convenient sources of advice” or information [15, 56, 58, 61] (p. 151) [64, 65] that help clinicians orientate treatment decisions [53, 55] and help decision making during treatment complications [61]. CPGs were considered to be “safety nets” to double check treatment decisions, especially when clinicians do not have access to MDTs for peer consultation about treatment plans [54] (p. 128). Many clinicians in one study found that CPGs help clinicians and patients reach agreement [59], and clinicians in another study felt they increased the confidence of clinicians when making treatment decisions [61]. CPGs were also thought to support clinicians’ legal defense, when adhered to [59, 61, 65] and that the successful defense of a clinician who had practiced CPG adherent care would act as a facilitator for uptake of CPG recommendations by others [61].

Positive clinician attitudes toward CPG recommendations were found to be a strong predictor of CPG adherence [58]. Just under two-thirds of clinicians in one study felt that specific CPG recommendations were balanced in terms of harms and benefits, that the specific CPGs in question were very good to excellent quality, and that CPGs were useful [15]. The clinicians in that study were confident about the CPGs under discussion [15]. The “multidisciplinary focus” of a particular CPG was considered an important factor when deciding to adhere to the CPG [65] (p. 365) and not being prescriptive was also considered a strength of that CPG [65]. Clinicians in one study reported that CPGs were part of their routine practice [59]. CPGs are perceived by clinicians to improve patient wellbeing and survival [59], and patient outcomes and quality of care [58, 65] or are intended to enhance the quality of patient care [15, 58, 64]. Clinicians also felt that CPGs reduced practice variation and increased the uniformity of care across disciplines, enabling consistent treatment communication with patients [54]. Half of the clinicians in one study felt that CPGs were intended to minimize healthcare costs [64].

## Discussion

This is the first review to identify clinicians’ attitudes toward, and perceived barriers to, and facilitators of, adherence to CPGs for active cancer treatments. This study specifically took into account the contributions of qualitative and quantitative research. The review identified four themes centered around negative attitudes and barriers, and another four focused on positive attitudes and facilitators.

These results highlight diversity in clinician views about CPGs. This may be related to variety in the quality of the guidelines, and associated evidence, being discussed in each study. One recurring theme was the lack of clinician trust in the evidence underpinning the CPGs. High-quality guidelines include details regarding the level of evidence underpinning each recommendation, identified through systematic review, or expert consensus, whereas poorer quality guidelines may not include that degree of detail [67–69]. This could explain clinician uncertainty regarding the evidence base, and the lack of outcome expectancy from adhering to CPGs, identified in the review. Infrequently updated CPGs may also contribute to these concerns [15], if they are underpinned by outdated evidence, as well as concerns about clinical trial publication bias [18].

Another clinician concern was that the evidence underpinning CPGs was based on clinical trials with cohorts of patients that were healthier or younger than the patients being treated, reflecting concern that this may invalidate the guidelines. While ideally CPGs would cater for all patient types, it is an inherent limitation that CPGs can only provide recommendations for patient cohorts, for which there is evidence to support treatments. This concern may be highlighting a need for greater clinical trial evidence regarding the efficacy of treatments in patients with poorer health status, older age, or comorbidities. The applicability of CPGs may be strengthened if real-world data sources (e.g., electronic health records) with more representative samples of patients [70] are incorporated into the evidence-base that underpins CPG recommendations, especially for patients who fit outside the study population of the original randomized trials.

In non-cancer specific literature, clinician experience or age were found to influence adherence to CPGs in general, with one review finding that less experienced clinicians were more likely to adhere to CPG recommendations than senior clinicians [11]. This factor was not identified in the present review, but has been found in a study looking at more general cancer related CPG adherence [71]. This may reflect the patient populations seen, with more experienced clinicians disproportionately treating more complex cases.

There were many attitudes, barriers, and facilitators identified in themes 1–4, 5, and 8 that overlapped with previously identified barriers and facilitators to general CPG adherence [7, 11, 13] (Table 6). This review also identified additional attitudes, barriers, or facilitators to active cancer treatment CPG adherence, specifically. Themes 6 and 7 solely identified factors that were specific to cancer treatment CPG adherence (Table 6).

**Table 6:** Comparison of previously identified factors and factors unique to cancer treatment CPG adherence

Previously identified factors [7, 11, 13] mirrored in this review	Factors identified in this review
**Theme 1 Barriers: concern over CPG content and currency of CPGs**	
CPGs are “Biased” [7] (p. 1459)	Some CPG recommendations are biased
CPGs lack “applicability to the practice population” [7] (p. 1460)	CPG are not always applicable to specific settings or feasible
	CPGs are not always clear
CPGs are “not easy to use” [7] (p. 1461)	CPGs can be hard to read
	Outdated CPGs, or slow to be updated
CPGs are “Oversimplified and cookbook medicine” [7] (p. 1461), 13	Some CPGs are perceived to lead to cookbook medicine that oversimplifies treatment decisions
CPGs are “Cumbersome and confusing” [7] (p. 1461)	Some CPGs are too complicated or complex to follow
CPGs are “Impractical and too rigid to apply” [7] (p. 1459), 13	Some CPGs are too rigid to apply to practice
	CPGs do not always take into account patient preferences or circumstances
CPGs lack “credibility by guideline authors” [7] (p. 1460)	Some CPGs were developed by people not engaged with clinical practice
	Concerned that CPGs are intended to cut costs
**Theme 2 Barriers: concern about the evidence underpinning CPGs**	
	CPGs underpinned by controversial evidence or a lack of evidence
	Clinical trial patient populations not reflective of the patients seen routinely by clinicians
	Contradicting CPGs that provide contradicting or controversial recommendations or advice
Clinicians “disagreed with a guideline due to differences in interpretation of the evidence” [7] (p. 1460)	Preference for own interpretation of the evidence over the synthesis of evidence in CPGs
	CPGs do not always take into account clinical experience
**Theme 3 Barriers: clinician uncertainty and negative perceptions towards CPGs**	
Clinicians reported concern about: A “lack of motivation” to change routines and “Inertia of Previous Practice” [7] (p. 1459)	Clinical equipoise and practice habits that differ to the CPG recommendations
A lack of “outcome expectancy” [7] (p. 1461)	A lack of outcome expectation of the CPG recommendations
That CPG “benefits were not worth patient risk, discomfort or cost” [7] (p. 1460)	Concerns about side effects associated with CPG recommendations
	Experience of patient adverse effects from CPG treatments
“A lack of self-efficacy” [7] (p. 1459)	Limited medical expertise to implement the CPG recommendation
	A perception that the CPG treatments are not necessarily appropriate for specific patients
A “lack of familiarity” and “awareness” of CPG [7] (p. 1459) 13	A lack of awareness of CPGs
“Reduced autonomy” [7] (p. 1460) [13], would decrease flexibility [7]	CPGs challenged clinician authority or autonomy
	Some CPGs limit the application of clinical judgment
A “lack of agreement” with the CPG [7] (p. 1460)	Clinicians disagreeing with specific CPG recommendations
	Limited experience with CPG recommended treatments
CPGs “will increased litigation or disciplinary action” [13] (p. 504)	Concerned that CPGs will expose them to litigation issues
**Theme 4 Barriers: organizational and patient factors**	
	Limited access to treatment services
	Treatment referral processes that are slow
	Referral processes that are unreliable
	Referral processes that are complex
	Surgeons’ hesitancy to refer patients to other clinicians
	A lack of support from organizational and clinical leadership
	CPG recommendations are not always cost effective
Clinicians reported barriers to adherence including “Patient factors” or characteristics [7] (p. 1459) [11], which may include factors like Patient preferences regarding treatment, Patient comorbidities and tumor specific characteristics [11]	Patient preferences regarding treatment choice
	Patient comorbidities and tumor specific characteristics
	The level of family support available to patients, and access to transport influences the treatment provided
	Family perceptions of or experiences of treatments were found to influence patient attitudes
	The age of the patient
	Concerns that costs of treatments or concern that adhering to CPG will increase healthcare costs, and other external barriers
	Poor accessibility to CPGs
**Theme 5 Facilitators: CPG accessibility and ease of use**	
	Having highly skilled clinicians with adequate expertise to implement the CPG is important
	CPGs should be thought of as guides
CPGs that are evidence based are more likely to be adhered to [11]	Some CPGs are considered good summaries of up-to-date evidence
Easy to use CPGs were more likely to be followed, if they don’t require specialized resources and can be easily trialed [11]	Some CPGs are considered easy to understand
	Some CPGs are considered flexible
	Some CPGs are considered implementable
	User-friendly formats were considered a strength of CPGs
	Some CPGs are developed in a timely manner
	CPGs should be updated regularly
	Adapting and revising CPGs to cater for local needs, and holding meetings about the revised CPG is an important factor
	Access to and availability of IT technology that integrates CPGs into the software used to record and order treatments, and provides feedback to clinicians is important
**Theme 6 Facilitators: endorsement and dissemination of CPGs along with adequate resources**	
	Clinician and clinical organizational support are important
	Collaboration between clinical disciplines in Multi-Disciplinary Teams (MDTs) is important
	Easy access to treatment services for patients is important
	CPG dissemination via medical college programs is important
	CPG endorsement by government research organizations is important
	CPG endorsement by medical colleges is important
	Recommendations by respected peers, or discussions with respected peers is important
	Symposia about CPGs are important
	Provision of emails or websites that summarized updated CPGs, or current clinical trials underpinning CPGs are important
	Access to treatment facilities and adequate resources to implement CPGs is important
	Audits and feedback are important
	Multidisciplinary clinical care pathways or MDT discussions increase awareness of CPGs
**Theme 7 Facilitators: awareness of CPGs and belief in their relevance**	
	High clinician awareness of CPGs
	Agreement with and support for CPG recommendations
	Confidence in CPGs was high when the guidelines were considered high quality
	Use of or compliance with CPGs was generally reported to be high
	CPGs should be “developed by credible individuals” and include lists of CPG committee members should be published
	Financial disincentives for surgeons who do not follow the guidelines
**Theme 8 Facilitators: CPGs support decision making, improve patient care, reduce clinical variation and reduce costs**	
CPGs were considered to be “helpful sources of advice” and information [13] (p. 504)	CPS are good, convenient sources of advice or information with unambiguous recommendations
CPGs were considered to be “good educational tools” for making treatment decisions [13] (p. 504)	CPS are considered to be good, useful and educational tools for making treatment decisions that help clinicians orientate treatment decisions
	CPGs help decision making during treatment complications, to double check treatment decisions, especially when clinicians don’t do not have access to MDTs
	CPGs reduced practice variation and increased the uniformity of care across disciplines
	CPGs help clinicians and patients to reach agreement
	CPGs increased the confidence of clinicians
	Support clinicians’ legal defense when they are adhered to
	CPG recommendations are balanced in terms of harms and benefits
	CPGs are clinically useful
	A “multidisciplinary focus” is important in CPGs
	Not being prescriptive is considered a strength of CPGs
	CPGs are part of routine practice
	CPGs improve patient wellbeing
	CPGs improve patient survival, outcomes and quality of care
CPGs were “intended to improve the quality of care” [13] (p. 504)	CPGs are intended to enhance the quality of patient care
CPGs were “intended to cut health care costs” [13] (p. 504)	CPGs are intended to minimize healthcare costs

These results highlight that adherence to cancer treatment CPG recommendations by oncology clinicians is influenced by multiple interlinked factors such as attitudes, practices, resouces available, and support provided by organisatoins [72]. It is important that cancer treatment CPG implementation strategies are multifaceted, and target patients, clinicians, organizations, and policy [10], taking into account the social and organizational structures that influence implementation, and ensuring that they are tailored to the local context [3]. These factors that are unique to cancer CPG adherence, also reflect the multi-disciplinary nature of modern cancer treatment, and the fact that many clinicians are involved in treating multiple different types of cancers and are therefore exposed to multiple CPGs. Similarly, they may reflect the fast pace development of cancer research, and the associated challenges with maintaining up to date CPGs, as well as the complexity of tailoring treatments to individual patient needs.

### Strengths and limitations

#### Limitations

This review restricted the inclusion criteria to studies regarding CPGs for active cancer treatment, which meant that CPGs focusing on an array of other key issues (e.g., prevention and screening, symptom management, psycho-social care, and palliative care) were excluded. The study also restricted the inclusion criteria to treating clinicians’ attitudes and perceived barriers and facilitators, which meant that studies that also included clinicians from other disciplines, such as psychologists and policy makers, were excluded if the attitudes of non-treating clinicians were not reported separately [73]. The review also restricted the criteria to only include studies published in English.

#### Strengths

This review consolidated knowledge about attitudes, barriers and facilitators that influence adherence to cancer treatment CPGs. While reviews conducted in past decades have identified barriers, facilitators or attitudes toward CPGs in general, this current systematic review is the first to combine all three facets, specifically targeting adherence to CPGs for active cancer treatment.

## Conclusion

We examined and thematized clinician attitudes to, and perceived barriers to and facilitators of, adherence to CPGs for active cancer treatment. The review has drawn attention to the many similarities and some differences in the factors associated with general CPG, and cancer treatment CPG, adherence. These findings will inform tailored implementation strategies to increase adherence to cancer treatment CPGs by overcoming specific barriers, considering the local context and utilizing the cancer treatment-specific facilitators, while being cognizant of the oncology-specific attitudes identified toward cancer treatment CPGs.

## Data Availability

Data sharing is not applicable to this article as no datasets were generated or analyzed during the current study.

## Appendix

**Table:** Table 7 PRISMA checklist

Section/topic	#	Checklist item	Reported on page #
Title			
Title	1	Identify the report as a systematic review, meta-analysis, or both.	1
Abstract			
Structured summary	2	Provide a structured summary including, as applicable: background; objectives; data sources; study eligibility criteria, participants, and interventions; study appraisal and synthesis methods; results; limitations; conclusions and implications of key findings; systematic review registration number.	3–4
Introduction			
Rationale	3	Describe the rationale for the review in the context of what is already known.	6–10
Objectives	4	Provide an explicit statement of questions being addressed with reference to participants, interventions, comparisons, outcomes, and study design (PICOS).	10
Methods			
Protocol and registration	5	Indicate if a review protocol exists, if and where it can be accessed (e.g., Web address), and, if available, provide registration information including registration number.	10
Eligibility criteria	6	Specify study characteristics (e.g., PICOS, length of follow-up) and report characteristics (e.g., years considered, language, publication status) used as criteria for eligibility, giving rationale.	11
Information sources	7	Describe all information sources (e.g., databases with dates of coverage, contact with study authors to identify additional studies) in the search and date last searched.	11–12
Search	8	Present full electronic search strategy for at least one database, including any limits used, such that it could be repeated.	12
Study selection	9	State the process for selecting studies (i.e., screening, eligibility, included in systematic review, and, if applicable, included in the meta-analysis).	11-12
Data collection process	10	Describe method of data extraction from reports (e.g., piloted forms, independently, in duplicate) and any processes for obtaining and confirming data from investigators.	12–13
Data items	11	List and define all variables for which data were sought (e.g., PICOS, funding sources) and any assumptions and simplifications made.	12–14
Risk of bias in individual studies	12	Describe methods used for assessing risk of bias of individual studies (including specification of whether this was done at the study or outcome level), and how this information is to be used in any data synthesis.	13
Summary measures	13	State the principal summary measures (e.g., risk ratio, difference in means).	13–14
Synthesis of results	14	Describe the methods of handling data and combining results of studies, if done, including measures of consistency (e.g., I^2^) for each meta-analysis.	13–14
Section/topic	#	Checklist item	Reported on page #
Risk of bias across studies	15	Specify any assessment of risk of bias that may affect the cumulative evidence (e.g., publication bias, selective reporting within studies).	13
Additional analyses	16	Describe methods of additional analyses (e.g., sensitivity or subgroup analyses, meta-regression), if done, indicating which were pre-specified.	
Results			
Study selection	17	Give numbers of studies screened, assessed for eligibility, and included in the review, with reasons for exclusions at each stage, ideally with a flow diagram.	15
Study characteristics	18	For each study, present characteristics for which data were extracted (e.g., study size, PICOS, follow-up period) and provide the citations.	15–16
Risk of bias within studies	19	Present data on risk of bias of each study and, if available, any outcome level assessment (see item 12).	15, 39
Results of individual studies	20	For all outcomes considered (benefits or harms), present, for each study: (a) simple summary data for each intervention group (b) effect estimates and confidence intervals, ideally with a forest plot.	15–24
Synthesis of results	21	Present results of each meta-analysis done, including confidence intervals and measures of consistency.	15–24
Risk of bias across studies	22	Present results of any assessment of risk of bias across studies (see Item 15).	15, 39
Additional analysis	23	Give results of additional analyses, if done (e.g., sensitivity or subgroup analyses, meta-regression [see Item 16]).	
Discussion			
Summary of evidence	24	Summarize the main findings including the strength of evidence for each main outcome; consider their relevance to key groups (e.g., healthcare providers, users, and policy makers).	25–27
Limitations	25	Discuss limitations at study and outcome level (e.g., risk of bias), and at review-level (e.g., incomplete retrieval of identified research, reporting bias).	27
Conclusions	26	Provide a general interpretation of the results in the context of other evidence, and implications for future research.	28
Funding			
Funding	27	Describe sources of funding for the systematic review and other support (e.g., supply of data); role of funders for the systematic review.	30

## Abbreviations

ACN: Australian Cancer Network
ASCO: American Society of Clinical Oncology
ASTRO: The American Society for Radiation Oncology
AUA: American Urological Association
cALND: Completion axillary lymph node dissection
CCOPGI: Cancer Care Ontario, Practice Guideline Initiative
COSA: Clinical Oncology Society of Australia
CPGs: Clinical Practice Guidelines
CRC: Colorectal cancer
CT: Can’t tell
DCIS: Ductal carcinoma in situ
IT: Information technology
MDTs: Multi-disciplinary teams
MDTM: Multi-disciplinary team meeting
MIBC: Muscle invasive bladder cancer
MMAT: Mixed Methods Appraisal Tool
MO: Medical oncologist
NCCN: National Comprehensive Cancer Network
NHMRC: National Health and Medical Research Council
NSCLC: Non-small cell lung cancer
PRISMA: Preferred Reporting Items for Systematic Reviews and Meta-Analyses
PROSPERO: PROSPERO: International prospective register of systematic reviews
RO: Radiation oncologist
SLNB: Sentinel lymph node biopsy
Surg: Surgeon
